# Higher ultra-processed food consumption is associated with poor nutritional quality but not with obesity in Israeli adults

**DOI:** 10.3389/fnut.2025.1586611

**Published:** 2025-06-03

**Authors:** Tal Shimony, Alina Rosenberg, Lital Keinan-Boker, Danit Rivka Shahar

**Affiliations:** ^1^Israel Center for Disease Control (ICDC), Israel Ministry of Health, Ramat Gan, Israel; ^2^The International Center for Health Innovation & Nutrition, Faculty of Health Sciences, School of Public Health, Ben-Gurion University of the Negev, Be’er-Sheva, Israel; ^3^School of Public Health, University of Haifa, Haifa, Israel

**Keywords:** NOVA, MABAT, nutrients, diet quality, 24 hour-recall

## Abstract

**Introduction:**

Ultra-processed foods (UPF) intake has increased in recent decades, coinciding with declines in diet quality, nutritional sufficiency, and rises in obesity and chronic diseases. Understanding its impact on health outcomes is crucial.

**Methods:**

This secondary analysis of Israel’s cross-sectional study, 2014–2016 National Health and Nutrition Survey includes 2,563 adults (25–64 years) with full dietary data. Data was obtained by a 24-hour recall based on an interview for dietary intake data, and anthropometric measurements such as weight, height, and waist circumference (WC). UPF consumption (percentage of total energy intake) was calculated classifying reported foods into four groups according to the NOVA method, based on their processing level. Diet quality was assessed by food group and nutrient intake by UPF consumption levels, while obesity was defined using BMI (≥30), relative fat mass, and waist circumference. Associations were tested using the chi-square test, the Cochran-Armitage trend test for dichotomous variables, and the Cochran–Mantel–Haenszel test for trend analysis across three or more categories. Additionally, logistic regression models were employed to account for potential confounders.

**Results:**

Participants were 46.8% men, average age 42.2 ± 11.1 years, with mean UPF consumption at 34.5% of total energy intake. Upper quartile UPF consumption was associated with younger age (*p* = 0.0015), low physical activity (*p* < 0.001) and smoking (*p* = 0.0162). Higher consumption of UPF was associated with high intake of energy, carbohydrates, saturated fat, and thiamine (*p* < 0.0001). It was also significantly linked to lower total intake of fat, protein, monounsaturated fatty acids, dietary fiber, folic acid, vitamin B_12_, vitamin C, and zinc. No significant association was found between UPF consumption and obesity (*N* = 1825).

**Discussion:**

UPF consumption is negatively correlated with nutrition quality but not with obesity among Israeli adults. Future obesity studies should thoroughly examine the etiological role of UPF. Furthermore, effective strategies should be developed to lower the level of processing in the food industry and to reduce the consumption of UPF.

## Introduction

In recent decades, obesity (defined as body mass index, BMI ≥ 30 kg/m^2^) rates have risen globally, ranging in adults, from 4.9% in Japan to 42.9% in the United States (1). Obesity is associated with numerous chronic diseases that place a heavy burden on healthcare systems (such as type 2 diabetes, cardiovascular disease, respiratory disease, kidney disease, liver disease, fertility impairment, cancer) and cause premature mortality (primarily from cardiovascular disease and cancer) (2). As such, obesity is often regarded as a disease in itself (3–5).

Along with the increase in obesity rates, significant diet changes were observed globally, with a decrease in natural foods in home preparation and an increase in the consumption of ultra-processed foods (UPF) (6). UPFs, as defined by the NOVA classification system, applied worldwide, are formulations of ingredients derived from very little or no whole foods, which use an industrialized method of preparation. The food classification is based on the nature, extent and purpose of food processing (6).

The world-wide consumption of UPFs has increased substantially in the last decades. In Canada, it doubled from 1938 to 2001, reaching 54.9% of daily kcal. In Brazil, the proportion of UPF rose from 18.7% of the total kcal in the food basket in 1987 to 26.1% in 2003 (7). Sweden experienced a 142% increase from 1960 to 2010 (8). Spain’s UPF consumption rose from 11% in 1990 to 31.5% in 2010 (9) contrasting with declines in Austria, Belgium, and Latvia (10). UPFs correlate with higher chronic disease rates, including cancer (11, 12), diabetes, and hypertension (13), inflammatory bowel disease (14), cardiovascular mortality (15), and all-cause mortality (16). Increasing UPF consumption is linked to poor diet quality, and nutrient deficiencies (17) along with obesity risk (18). These may impact directly healthy growth and development in children and the incidence of chronic diseases (11–14, 19).

Despite the well-documented health risks associated with UPFs, including their role in chronic disease prevalence, the impact on nutritional quality is especially concerning. In Israel, the Ministry of Health’s dietary guidelines advocate reducing UPF intake, but lack comprehensive data on its consumption. This information is required for identifying high-risk groups and for developing and evaluating related intervention programs.

The study aims to address these gaps by exploring the extent of UPF consumption among Israeli adults and its specific impacts on dietary quality, nutrient sufficiency, and obesity prevalence among the Israeli population. By focusing on these aspects, we seek to provide a deeper understanding of how UPFs affect health beyond the scope of obesity.

## Methods

### Study design and population

**Figure 1:**
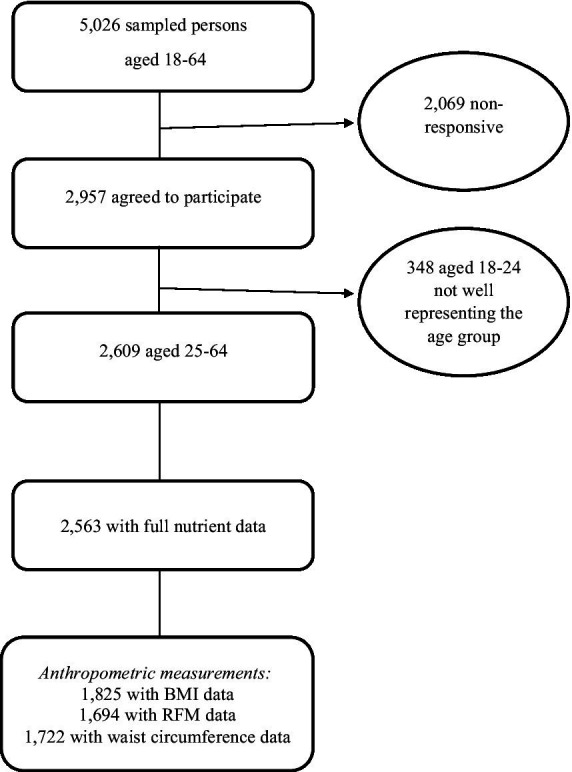
Creation of the final analytical data set from Rav MABAT adults survey 2014–16. BMI, body mass index; RFM, relative fat mass.

The Rav MABAT^1^ Adults survey, a national Israeli health and nutrition survey, was conducted between 2014 and 2016 (20). Participants included community-dwelling women and men of all religions. New immigrants were included in the survey only if they had been residing in Israel for at least 6 months prior to its execution. Participants were categorized by ethnic group based on Population Registry data, to account for differences in lifestyle, nutrition, and morbidity rates (21). For purposes of the current study, analyses were performed on 2,563 adults aged 25–64 with full nutrient data (Figure 1).

Trained interviewers conducted a single face-to-face interview with the interviewees in their homes, using the constructed survey questionnaire. The interviews were conducted in either Hebrew or Arabic, based on ethnic group distribution. Demographic and lifestyle data were collected, as well as dietary assessment and anthropometric measurements.

The Ethics Committee of the Ministry of Health approved the survey, and prior to the start of the interview, each interviewee signed an informed consent form (certificate number: MOH 043-2012).

### Foods classification according to the degree of processing

According to the NOVA method, foods are classified into four categories: (1) unprocessed or minimally processed foods; (2) processed culinary ingredients; (3) processed foods combining levels 1 and 2; and (4) ultra-processed foods and beverages characterized by high number of ingredients and industrialized methods of preparation (6).

### Dietary assessment

Dietary intake assessment was performed using a 24-hour food recall, a method widely accepted by the United States Department of Agriculture (USDA), based on a personal interview with a skilled interviewer. The interview includes several steps of questioning: presentation, creating a quick food list without details, asking about forgotten food items, collecting detailed information on the food items consumed, summarizing and repeating the food items reported. The questioning method makes it possible to obtain detailed and accurate information on food consumption from the previous day. All participants filled out the 24-hour dietary recall. Data entry into “Tzameret,” a designated software based on an Israeli database of food products, was conducted by trained personnel, predominantly registered dietitians, under a strict quality assurance protocol. The database is derived from several sources, such as the USDA food database, local manufacturer data, and laboratory nutrients tests, and is updated for the survey period. Macro- and micronutrient daily consumption was calculated. All 2,700 food items reported in the survey were categorized according to NOVA classification.

### Anthropometric measurements

Weight, height, and waist circumference measurements were measured using the following simple tools (20). Weighing was performed using an analog scale suitable for weighing up to 130 kg, with accuracy of 1 kg; height was measured using a spring coil measuring tape. A rigid aluminum angle was used to determine the intersection of the meeting point of the top of the head with the wall/door, and stickers were used to mark the meeting point. Waist circumference was measured using a flexible tape that allows for a measurement of a maximum of 1.5 m. These indices were used to calculate obesity based on proxy measures such as body mass index (BMI) (weight in kg divided by height in m, squared), relative fat mass (RFM) (based on a ratio of height and waist measurements in meters^2^), and abdominal obesity (waist circumference in cm). Obesity was defined as BMI ≥ 30 kg/m^2^ (22), male RFM ≥ 30%, female RFM ≥ 40% (23), male waist circumference ≥102 cm, and female waist circumference ≥88 cm (24). Pregnant women were excluded from the anthropometric indices analyses. BMI, RFM, and waist circumference data was available for 1,825, 1,694, and 1,722 sampled persons, respectively.

### Physical activity

Respondents reported the frequency (minutes per week) of performing various physical activities during their last typical week. The activities included walking, running, cycling, swimming, aerobic exercise, body shaping exercise like yoga or Feldenkrais, weight lifting, ball games, and martial arts.

For purposes of the current study, Physical activity data were collected from 2,537 respondents and categorized based on compliance with World Health Organization (WHO) recommendations: at least 150 min of moderate-intensity aerobic physical activity throughout the week or at least 75 min of vigorous-intensity aerobic physical activity throughout the week or an equivalent combination of moderate- and vigorous-intensity activity to sufficiently meet the physical activity level (25).

### Smoking

Respondents reported their smoking status (current smoker/past smoker/never smoked), which was defined in two different ways: the first includes current+past smoking/never smoking and the second includes current smoking/past+never smoking.

### Poverty line

The poverty line was calculated based on household income and the number of people living in each household. Data on poverty income was based on the 2014 Poverty Report of the National Insurance Institute, Israel (26).

### Statistical analysis

All statistical analyses were performed using SAS software. Statistical significance was set at *α* < 0.05 for all tests. Continuous variables were presented as an average with standard deviation [SD], median, and interquartile range. Categorical variables are presented as absolute numbers and percentages.

UPF consumption was characterized as the average percentage of total energy intake attributed to UPFs, presented in quartiles. Key food items and groups contributing the most to UPF and beverage consumption were identified.

The four levels of UPF intake were compared by demographic and nutritional variables using the chi^−^square test, Cochran-Armitage trend test for testing dichotomic variables, and the Cochran–Mantel–Haenszel statistics test for trend analysis across three or more categories. The average percentage of macro- and micronutrient intake by quartiles of UPF intake was calculated using the chi-square test and the Jonckheere-Terpstra test for trend testing. The association between UPF consumption and different obesity measures was examined, first in univariable (chi-square test) and then in multivariable logistic regression models, using backward elimination method based on *p*-values, adjusted for potential covariates (statistically significant variables in the univariable analyses). These included age, education, physical activity, ethnic group and poverty status for the BMI obesity definition; sex, age, marital status, physical activity, education, poverty status, and ethnic group for the RFM obesity definition; and sex, age, marital status, education, poverty status, physical activity, and ethnic group for the waist circumference obesity definition.

## Results

The current data analysis included 2,563 participants aged 25–64. 46.8% of the survey population were men, 81.8% were Jews/others. 36.8% had academic education (first academic degree to third academic degree) and 25.3% were current smokers. The mean percentage of UPF consumption energy of the total daily energy intake was 34.5%. Unprocessed or minimally processed foods accounted for 15.2%, processed culinary ingredients for 5%, and processed foods for 45.2%.

Table 1 describes the characteristics of UPF consumers in Israel by quartiles (1 = lowest; 4 = highest). Participants at the highest quartile of UPF consumption were younger (29.1% ages 25–34, 21.6% ages 55–64, *p* = 0.0015), reported lower physical activity (27.1% not with accordance to WHO recommendations, 20.4% with accordance to WHO recommendations, *p* < 0.001), and had higher prevalence of current smoking (29.3% smokers, 23.3% non-smokers, *p* = 0.0162). Using an alternative classification of smoking status (current+past smoking/never smoking) showed a similar trend (p-for trend = 0.0147) but this was not statistically significant.

**Table 1:** Characteristics of consumers of ultra-processed food (UPF), Israeli adults ages 25–64.

Variables		*n*	Mean UPF consumption (% of total energy)	Percent in UPF quartiles				*p*-value (for quartile differences)	P for trend
				1st Quartile (<18.4%)	2nd Quartile (18.4–32.71%)	3rd Quartile (32.72–48.73%)	4th Quartile (>48.73%)		
Sex	Males	1,200	35.7	23.1	25.4	24.5	27.0	0.0588	0.02
	Females	1,363	33.6	26.7	24.7	25.5	23.2		
Age [y]	25–34	746	36.9	22.4	22.0	26.5	29.1	0.0015	0.0015
	35–44	804	35.2	22.5	26.0	25.9	25.6		
	45–54	563	32.5	28.6	26.5	23.6	21.3		
	55–64	450	32.0	29.3	26.4	22.7	21.6		
Ethnic group	Jews and others	2,097	34.9	24.9	24.6	24.5	25.9	0.099	0.1461
	Arabs	466	33.0	25.3	26.8	27.3	20.6		
Marital status	Married	1,923	34.0	25.3	25.9	25.2	23.7	0.0532	0.0428
	Single/divorced/widowed/separated	635	36.1	24.4	22.4	24.4	28.8		
Education	12 years or less	1,003	34.5	26.4	23.5	24.1	25.9	0.549	0.5492
	13 years or more—not academic	580	35.5	23.3	25.9	25.9	25.0		
	13 years or more—academic	921	33.9	24.5	26.4	25.5	23.5		
Poverty status	Above poverty line	1,608	34.7	23.9	25.2	26.0	24.9	0.3796	0.6978
	Under poverty line	425	35.7	25.7	22.1	24.5	27.8		
Physical activity^a^	In accordance with the WHO recommendations	809	31.6	31.3	24.2	24.1	20.4	<0.001	<0.001
	Not in accordance with the WHO recommendations	1,689	36.0	22.0	25.5	25.5	27.1		
Smoking status	Current smoker	641	36.6	22.3	24.8	23.6	29.3	0.0162	0.0073
	Not a current smoker	1,981	33.8	26.0	25.2	25.5	23.3		
Ever smoking	Current and past smoking	1,103	35.5	23.4	23.9	26.0	26.7	0.0958	0.0147
	Never smoking	1,429	33.7	26.3	25.9	24.4	23.4		

a Adults aged 18–64 years should do at least 150 min of moderate-intensity aerobic physical activity throughout the week, or do at least 75 min of vigorous-intensity aerobic physical activity throughout the week, or an equivalent combination of moderate- and vigorous-intensity activity.

Table 2 presents the daily total energy and the percent of energy intake from UPFs consumption of selected food groups. For each food group, the percent of UPF of the group’s total consumption is presented. The consumed UPFs varied widely between food groups, ranging from 136 kcal, 0.6% in fruits, and 107 kcal, 3.3% in vegetables, to 100% in sugar substitute sweetened soft drinks. Almost 90% of baked goods, based either on white or whole wheat flour, were considered UPFs. As for legumes, 29.1% were identified as UPFs, mostly including industrial spreads like hummus.

**Table 2:** Daily total energy and percent energy from UPFs by selected food groups, Israeli adults ages 25–64.

Food group	Number of consumers	Total energy (kcal)				% Energy of UPF
		Mean	SD	Median	IQR	
Sugar sweetened soft drinks	861	181	167	133	79,222	82.9
Sugar substitute sweetened soft drinks	214	13	20	7	5,12	100
Baked foods (breads)—white flour	1,501	320	423	260	161,411	89.8
Baked foods (breads) whole grain	827	236	188	183	110,312	86.9
Dairy—sugar substitute sweetened	30	73	49	57	56,59	94.6
Dairy—sugar sweetened	328	150	96	132	94,197	66.7
Dairy—not sweetened	2,048	163	150	123	60,217	16.5
Sweets	1,870	153	200	88	35,201	44.2
Processed meat	212	103	116	59	37,134	99.5
Meat	684	261	322	171	92,325	3.2
Poultry, turkey	1,187	232	177	190	124,293	3.2
Fish	552	212	195	164	73,298	1.6
Legumes	834	143	132	97	43,193	39.1
Nuts and seeds	854	175	196	116	54,116	16.8
Vegetables	2,325	107	98	79	39,144	3.3
Fruits	1,337	136	117	96	59,176	0.6

Table 3 describes the intake of selected macro- and micronutrients by quartiles of UPF consumption. Higher UPF consumption was associated with higher intake of energy, carbohydrates, saturated fat (SFA) and thiamine (*p* < 0.0001), and lower intake of total fat (*p* = 0.0059), protein, monounsaturated fat (MUFA), dietary fiber, folic acid, vitamin B_12_, vitamin C and zinc (*p* < 0.0001). Calcium and polyunsaturated fat (PUFA) intake were higher among participants in the highest UPF quartile, but these differences were not statistically significant. However, the *p*-values for trends were significant for both 0.0201 and 0.0135, respectively.

**Table 3:** Nutrients intake by quartiles of UPF consumption, Israeli adults ages 25–64.

Nutrient (*n* = 2,563)	Ultra-processed food consumption (mean ± SD)				p-value	P for trend
	1st Quartile (<18.4%)	2nd Quartile (18.4–32.71%)	3rd Quartile (32.72–48.73%)	4th Quartile (>48.73%)	
Total energy, kcal	1,481 ± 785	1,644 ± 790	1,677 ± 777	1,647 ± 813	<0.0001	<0.0001
Protein (% of total kcal)	20.2 ± 8.1	18.7 ± 6.5	17.0 ± 5.6	15.0 ± 5.0	<0.0001	<0.0001
Carbohydrates (% of total kcal)	43.1 ± 13.1	45.4 ± 10.3	48.9 ± 9.8	51.9 ± 10.4	<0.0001	<0.0001
Total fats (% of total kcal)	33.9 ± 9.9	33.7 ± 8.8	32.6 ± 8.1	32.3 ± 9.1	0.0059	0.0007
Saturated fats (% of total kcal)	9.3 ± 4.3	9.8 ± 3.8	10.1 ± 4.0	10.8 ± 4.5	<0.0001	<0.0001
Poly un-saturated fats (% of total kcal)	7.9 ± 4.5	8.0 ± 3.9	7.9 ± 3.5	8.1 ± 4.0	0.1056	0.0201
Mono-saturated fats (% of total kcal)	14 ± 5.4	13.3 ± 4.6	12.1 ± 4.2	11.1 ± 4.2	<0.0001	<0.0001
Dietary-fiber (g/1,000 kcal)	12.9 ± 6.7	12.9 ± 6.2	12.5 ± 6.3	11.5 ± 6.6	<0.0001	<0.0001
Folic acid (mcg/1,000 kcal)	182 ± 105	174 ± 121	167 ± 94	154 ± 79	<0.0001	<0.0001
Thiamin (mg/1,000 kcal)	0.7 ± 0.2	0.7 ± 0.3	0.7 ± 0.3	0.8 ± 0.4	<0.0001	<0.0001
Vitamin B12 (mcg/1,000 kcal)	2.7 ± 4.0	2.2 ± 2.0	2.3 ± 5.6	1.7 ± 1.8	<0.0001	<0.0001
Vitamin C (mg/1,000 kcal)	74.0 ± 81.0	63.2 ± 73.8	53.7 ± 56.1	39.6 ± 42.2	<0.0001	<0.0001
Calcium (mg/1,000 kcal)	376 ± 197	396 ± 242	387 ± 194	404 ± 210	0.0878	0.0135
Iron (mg/1,000 kcal)	6.4 ± 2.6	6.4 ± 2.4	6.5 ± 2.6	6.5 ± 2.5	0.2576	0.0745
Zinc (mg/1,000 kcal)	6.1 ± 5.8	5.4 ± 2.0	5.0 ± 2.2	4.5 ± 1.9	<0.0001	<0.0001
Sodium (mg/1,000 kcal)	1,707 ± 689	1,744 ± 635	1,713 ± 654	1,657 ± 622	0.1434	0.2333

**Figure 2:**
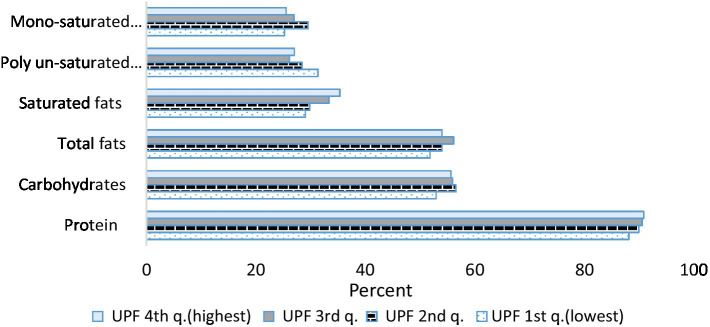
Percent of macro-nutrients consumption according to the reference value by consumption of ultra-processed foods. Israeli adults ages 25–64, 2014–16. For proteins, carbohydrates and total fats the reference value is the DRI (Dietary Reference Intake): carbohydrates make up 45 to 65% of total daily calories, total fat—20 to 35% of total daily calories and protein—10 to 35% of total daily calories. For saturated fats, mono-unsaturated fats and poly-unsaturated fats the reference value is the Israel Ministry of Health and the IOM (Institute of Medicine, USA): saturated fats make up 7 to 10% of total daily calories, mono-unsaturated—10 to 13% of total daily calories and poly-unsaturated—8 to 12% of total daily calories.

**Figure 3:**
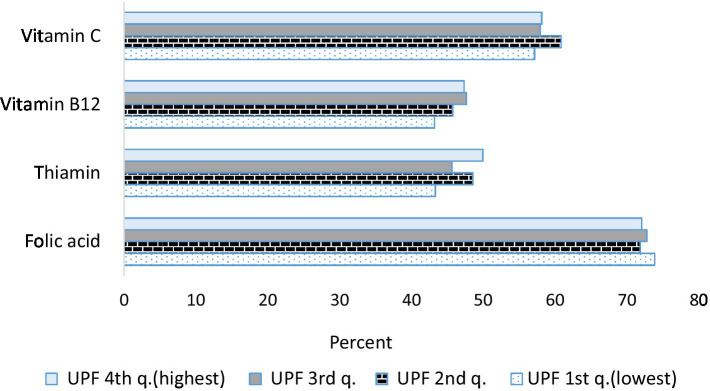
Percent of selected vitamin consumption less than the reference value (estimated average requirements—EAR) by consumption of ultra-processed foods. Israeli adults ages 25–64, 2014–16. For adults aged 19 + years, EAR for vitamin C is 75 mg/d for males and 60 mg/d for females, EAR for vitamin B_12_ is 2 mcg/d, EAR for thiamin is 1 mg/d for males and 0.9 mg/d for females, EAR for folic acid is 320 mcg/d.

**Figure 4:**
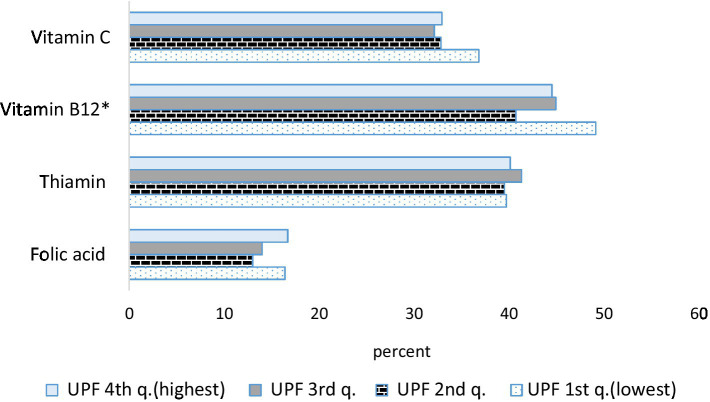
Percent of selected vitamin consumption with reference to international recommendations (recommended dietary allowance—RDA) of the overall diet according to the consumption of ultra-processed foods. Israeli adults ages 25–64, 2014–16. *p < 0.05. For adults aged 19 + years, RDA for vitamin C is 90 mg/d for males and 75 mg/d for females, RDA for vitamin B_12_ is 2.4 mcg/d, RDA for thiamin is 1.2 mg/d for males and 1.1 mg/d for females, RDA for folic acid is 400 mcg/d.

Figures 2–4 describe the consumption of selected macronutrients and vitamins (foods and beverages only) in accordance with the dietary guidelines by quartiles of UPF consumption in the survey population. No association was found.

Table 4 presents the association between UPF consumption and obesity according to three indices: BMI, RFM, and abdominal obesity (waist circumference). No association was found between obesity and UPF intake, even after adjusting for additional variables, such as sex, age, ethnic group, education, physical activity, and poverty status. Similar results were found in sensitivity analysis where obesity by BMI was defined as BMI ≥ 35 kg/m^2^ (data not shown).

**Table 4:** Crude and adjusted analyses of the association between the dietary contribution of percent UPFs and indicators of obesity, Israeli adults ages 25–64.

Obesity measure		Ultra-processed foods quartiles (% of total energy)				P-value	1	2	3	4
Model 1: Obesity by BMI^a^ (*N* = 1,825)						
	Mean BMI (SD)	26.1 (5.1)	26.0 (4.8)	25.7 (5.1)	25.6 (5.2)	0.291
	Prevalence (%)	20.8	19.0	17.6	19.1	0.6731
	Crude, OR (95% CI)	1	0.89 (0.642–1.235)	0.813 (0.584–1.133)	0.901 (0.648–1.252)	0.6811
	Model 1^b^, OR (95% CI)	1	0.89 (0.612–1.294)	0.793 (0.542–1.159)	0.882 (0.603–1.289)	0.6969
Model 2: Obesity by RFM^c^ (*N* = 1,694)						
	Males: mean RFM (SD)	27.1 (5.6)	26.6 (5.1)	26.3 (5.1)	26.8 (5.2)	0.2898
	Females: mean RFM (SD)	37.3 (5.6)	37.4 (5.8)	36.4 (6.8)	36.5 (6.1)	0.3145
	Prevalence (%)	30.8	31.8	28.5	27.5	0.492
	Crude, OR (95% CI)	1	1.056 (0.787–1.417)	0.891 (0.661–1.2)	0.853 (0.631–1.153)	0.4514
	Model 2^d^, OR (95% CI)	1	1.122 (0.789–1.596)	0.942 (0.66–1.344)	0.956 (0.667–1.371)	0.7528
Model 3: Abdominal obesity by waist circumference^e^ (*N* = 1,722)						
	Males: mean WC (SD)	95.8 (14.4)	94.6 (12.2)	94.2 (12.5)	95.1 (13.6)	0.3921
	Females: mean WC (SD)	85.2 (12.6)	85.1 (12.1)	83.8 (13.9)	83.5 (13.7)	0.2551
	Prevalence (%)	52.4	50.0	45.2	48.1	0.1988
	Crude, OR (95% CI)	1	0.913 (0.697–1.196)	0.747 (0.569–0.95)	0.84 (0.64–1.103)	0.1829
	Model 3^f^ OR (95% CI)	1	0.841 (0.599–1.182)	0.788 (0.562–1.103)	0.798 (0.566–1.126)	0.5036

a BMI ≥ 30.

b Adjusted for age, education, physical activity and poverty status (*N* = 1,466).

c RFM ≥ 30 for males and RFM ≥ 40 for females.

d Adjusted for age, education, poverty status, sex and ethnic group (*N* = 1,365).

e Waist circumference ≥102 for males and ≥ 88 for females.

f Adjusted for age, education, physical activity, sex and ethnic group (*N* = 1,383).

## Discussion

The aim of the present study was to describe the consumption of UPF in Israel based on a representative survey among 2,563 participants aged 25–64 of the Israeli National Health and Nutrition Survey conducted in 2014–16. We investigated the prevalence of UPF consumption and its association with diet quality, nutrient intake, and obesity. Our findings indicate that UPFs account for 34.5% of total energy consumption in Israel.

The findings regarding UPF consumption align with the Israeli survey conducted in 2010 (12) as well as with reports from other Western countries (13, 15, 27–32). The proportion of UPFs in energy intake is higher in the United States (55%) and England (54.3%) (31, 33). In other countries, the total energy consumption attributable to UPFs as a percentage of total energy ranges from 4 to 47%, with low values found in Italy and China (13, 15, 27–29, 32). UPF consumption decreased with age (31, 34–37) and was linked to physical activity [negative association] and smoking [positive association], as seen in France, Canada, and Korea (34, 36, 37).

The study findings indicated that sweetened beverages are widely consumed in Israel and thus contribute significantly to the consumption of ultra-processed foods. The adverse health outcomes of sweetened beverages are well documented (38). Interestingly, baked goods such as bread—even whole-grain varieties, which are often regarded as healthy staples—are commonly manufactured using industrial methods and ingredients, classifying them as ultra-processed foods. The substantial contribution of sweetened beverages and bread to UPF consumption has been well-documented also across numerous countries (39–42). It remains crucial to encourage the public to substitute sweetened beverages with water and explore ways to enhance the quality of ready-made baked goods and bread, making them less ultra-processed.

In the current survey, UPF consumption was negatively associated with nutritional quality in terms of micro- and macronutrients intake, as also seen in a French survey performed among adults aged 45 years and older (16) and among Portuguese adults and the elderly (43). A study conducted in Brazil found that UPFs contain fewer vitamins and minerals compared to natural or minimally processed foods (44). UPFs are known to be less satiating, more hyperglycemic, hyper palatable, and hence, encourage overconsumption (42, 45).

Calcium intake, in the current study, was not related to UPF intake. A primary source of dietary calcium is dairy products, which in Israel undergo partial ultra-processing: 67% of dairy products consumed are sugar sweetened and 95% of sugar substitutes dairy products consumed are UP, compared to only 16.5% of unsweetened dairy products. Remarkably, ultra-processed dairy items retain comparable calcium content to their minimally processed counterparts, resulting in a lack of variance in calcium intake across different levels of UPF consumption. These findings align with those derived from the National Health and Nutrition Examination Survey data in the United States (35) and are consistent with a study in Brazil, which attributed the absence of association between UPFs and low calcium intake to the fortification of foods with calcium and the utilization of calcium-rich cheeses in pre-prepared food products (44).

We showed that sodium intake per 1,000 kcal did not differ between UPF quartiles. These findings are in accord with studies conducted in the United States, Canada, and France, which found no association between sodium intake and UPF consumption (27, 30, 42). Likewise, an Israeli study examined sodium consumption by measuring sodium excretion in urine. The findings suggested that sodium intake is influenced by daily energy intake, and when this was adjusted to energy intake, the differences in sodium intake were eliminated (46), further stressing the possibility that adjustment for energy intake accounts for sodium intake differences. In contrast, a Korean study found an inverse relationship between sodium intake and UPF consumption. The researchers attributed this finding to the consumption of local foods that are naturally rich in sodium (37).

In the present study, the relationship between UPF intake and obesity was examined using three different indices: BMI, RFM and WC. In contrast with findings from other countries, none of these indices was related to UPF intake. Studies from other countries have shown a linear association between UPF intake and obesity. These include England (31, 47), France (34), Canada (36, 37), Brazil (48), and even China (28).

UPF consumption was related in the last decades to poor nutrition quality and obesity, as well as other chronic diseases (49–51). We found no association between UPF consumption and obesity, similar to a few other studies (52, 53). This may stem from different classifications, different amounts of added ingredients in UPFs and differences in obesity rates. Selection and information biases may be present, and publication bias may also play a role.

Interestingly, the current survey observed lower total energy consumption among individuals classified as having obesity based on BMI. This discrepancy between reported energy intake and obesity status suggests the possibility of underreporting food intake, a common tendency among individuals with overweight and obesity, which may lead to an underestimation of actual energy consumption (54–56). Variations in food intake reporting could confound the association between UPF intake and obesity. Furthermore, reversed causality may be at play: Individuals with obesity in the survey may have actively reduced their UPF consumption in an effort to lose weight, but this change was not yet reflected in the study.

### Study limitations and strengths

The MABAT nutrition survey is a classic cross-sectional study where exposure and outcome are assessed simultaneously, limiting causative inference. Additionally, food recall data was processed for 1 day only, which may not correctly capture the habitual consumption of the participants, despite being considered representative on the national intake level. This limitation might explain the lack of association between UPF consumption and obesity prevalence. To better characterize food consumption over time, it is advisable to perform several food recalls, on different weekday and weekend days or use other acceptable methods.

Furthermore, the response rate for anthropometric measurements was incomplete, with BMI calculated for about 72% of participants. To ensure that the body structure of the measured population did not differ from that of the non-measured population, BMI values were also calculated based on self-reported height and weight for all participants. No significant difference in mean BMI was found between the two groups, suggesting that the anthropometric measurements used to calculate obesity indices were unbiased and accurately represented the overall study population. Moreover, all obesity measures used were proxy measures commonly applied to assess the relationship between body composition, morbidity, and mortality. Despite this, three different measures were used, with similar outcomes. With respect to the NOVA classification system, which is widely used and has gained popularity in nutritional research, it is important to acknowledge that not all of its underlying assumptions have been scientifically validated.

Our study has several strengths. The survey, conducted with a representative sample of the general population in Israel, gathered information through face-to-face interviews and direct anthropometric measurements. Structured interviews with quality control procedures and skilled surveyors ensured the accuracy of general, nutritional, and anthropometric data. Additionally, respondents reported their food consumption for the day prior to the interview, with the data meticulously coded into 2,700 distinct products, ensuring a high level of precision in food classification according to the NOVA method.

## Conclusion

In the present study, a high-level of UPF consumption was associated with poor nutritional quality but not with obesity. There is a need to further examine UPF consumption and overweight and obesity in a prospective follow-up. UPF consumption was related to lower diet quality in terms of vitamins, and minerals intake. This can imply that people who frequently consume higher amount of UPF should be conscious about their intake of nutrients. The results indicate that in food groups such as dairy products and bread, the majority of items consumed in Israel are categorized as ultra-processed. While these foods are often recommended as part of a balanced diet due to their essential nutrients—such as calcium in dairy products and B vitamins in bread—it is important to encourage the selection of minimally processed options within these groups. At the same time, fostering collaboration with the food industry should be a priority to reduce the processing levels of these vital food items, which are integral to a nutritious and balanced diet.

## Data Availability

The data analyzed in this study is subject to the following licenses/restrictions: The data described in the manuscript is based on a national survey in collaboration with the Central Bureau of Statistics (CBS) under strict data security regulations. It is expected that these data will be made available to the general public by the CBS following de-identification, through designated research platforms. Requests to access these datasets should be directed to Naama Rotem, naama@cbs.gov.il.

## Footnotes

^1^MABAT is the Hebrew acronym for “Health and Nutrition Status.”

^2^Mature males: 64–20 × (height/waist circumference), Mature females: 76–20 × (height/waist circumference).
